# Rosai‐Dorfman disease presenting with solitary liver mass without lymphadenopathy: A case report

**DOI:** 10.1002/ccr3.4709

**Published:** 2021-08-25

**Authors:** Yousef Roosta, Ali Esfahani, Amir Vahedi, Kosar Tarvirizadeh, Sadegh Asoubar, Behdad Boroofeh, Roshan Dinparast, Farhad Behzadi, Mortaza Raeisi, Mohammadreza Mohammad Hosseiniazar

**Affiliations:** ^1^ Department of Internal Medicine School of Medicine Urmia University of Medical Sciences Urmia Iran; ^2^ Hematology and Oncology Research Center Tabriz University of Medical Sciences Tabriz Iran; ^3^ Department of Clinical Pathology Tabriz University of Medical Sciences Tabriz Iran; ^4^ Medicine Faculty Tabriz University of Medical Science Tabriz Iran

**Keywords:** histiocytosis, hypodense liver mass, lymphadenopathy, Rosai‐Dorfman Disease

## Abstract

Rosai‐Dorfman disease (RDD), as a lymphoproliferative disorder with unknown etiology, is commonly identified with systemic clinical manifestations in various organs. In this case study, RDD occurrence was reported with an exceedingly liver mass.

## Graphical abstract

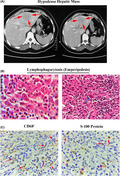

## 1 INTRODUCTION

An adult woman was referred with abdominal pain, fever, orthostatic hypotension, night sweats, and anemia. Following the primary diagnosis, hypodense mass was observed in the liver during the CT scan. The lymphophagocytosis and positive results for CD68 and S‐100 markers further confirmed an RDD. Finally, the patient underwent a hepatectomy.

Rosai‐Dorfman disease (RDD), also termed as non‐Langerhans cell histiocytosis, is a rare idiopathic macrophage‐related disorder with a heterogeneous entity, which is most likely concerning autoimmune or malignant diseases. RDD is characterized by the accumulated histiocytosis in various organs, more commonly in lymph nodes, skin and soft tissues, upper respiratory tract, and sinuses with less common occurrence in the central nervous system.^1^ Although the exact etiology of the disease is partially known, the patients mostly present with a localized or disseminated RDD with symptoms including fever, leukocytosis, and painless cervical lymphadenopathy (CLA).^2^ In its recent revised classification in 2016, the Histiocyte Society has classified RDD into the following subtypes: familial RDD, classical RDD, extranodal RDD, neoplasia‐associated RDD, and immune disease‐associated RDD.^3^ Of these, classic RDD presents with a painless CLA and enlarged or swollen lymph nodes in children and young adults, which in part is more common in men (58%) with a benign self‐limiting course.^4^ Older age and underlying immunologic abnormalities (eg, autoimmune hemolytic anemia, Wiskott‐Aldrich syndrome, glomerulonephritis, and rheumatoid arthritis) are directly associated with a complicated form of the disease and chronic relapsing course.^5^

Recently, Goyal et al. evaluated the clinicopathological features of RDD.^4^ According to the results, of 64 patients with a mean age of 50 years, the most common presentation was subcutaneous masses (40%) with extranodal disease (92%) in comparison with nodal disease (classical form, 8%). In general, RDD is defined as a self‐limited disease; however, it can be also permanent or seldom a life‐threatening condition.^6^ The diagnosis of RDD mainly relies on histology and immunohistochemistry (IHC) assessments along with diagnostic imaging. IHC staining of the histiocytes are positive for S‐100, CD68, and CD163, while CD1a^+^ cells are detected for Langerhans cell histiocytosis.^6^ In some forms of RDD, the mutation landscape of *MAP2K1*, *KRAS*, *NRAS*, and *ARAF* genes in lesional tissues was also reported.^7^ Although a large portion of patients experience spontaneous remission after a few months or years without receiving treatment, the rarity and unpredictability of RDD render a challenging treatment modality. Despite this, therapeutic measures are best tailored to RDD patients’ clinical manifestations for symptomatic form with critical organ involvement. In this regard, the therapeutic approaches compromise surgical intervention (resection/debulking), systemic corticosteroids (eg, prednisone 40–70 mg per day), chemotherapy, immunomodulatory therapy (INF‐α and rituximab), imatinib, sirolimus, thalidomide, and radiotherapy.^7^ However, the development of a recurrent form of the disease was also occasionally reported.^4^ This article has introduced a rare case report of RDD with a solitary liver mass lesion without lymphadenopathy involvement.

## 2 CASE PRESENTATION

**FIGURE 1:**
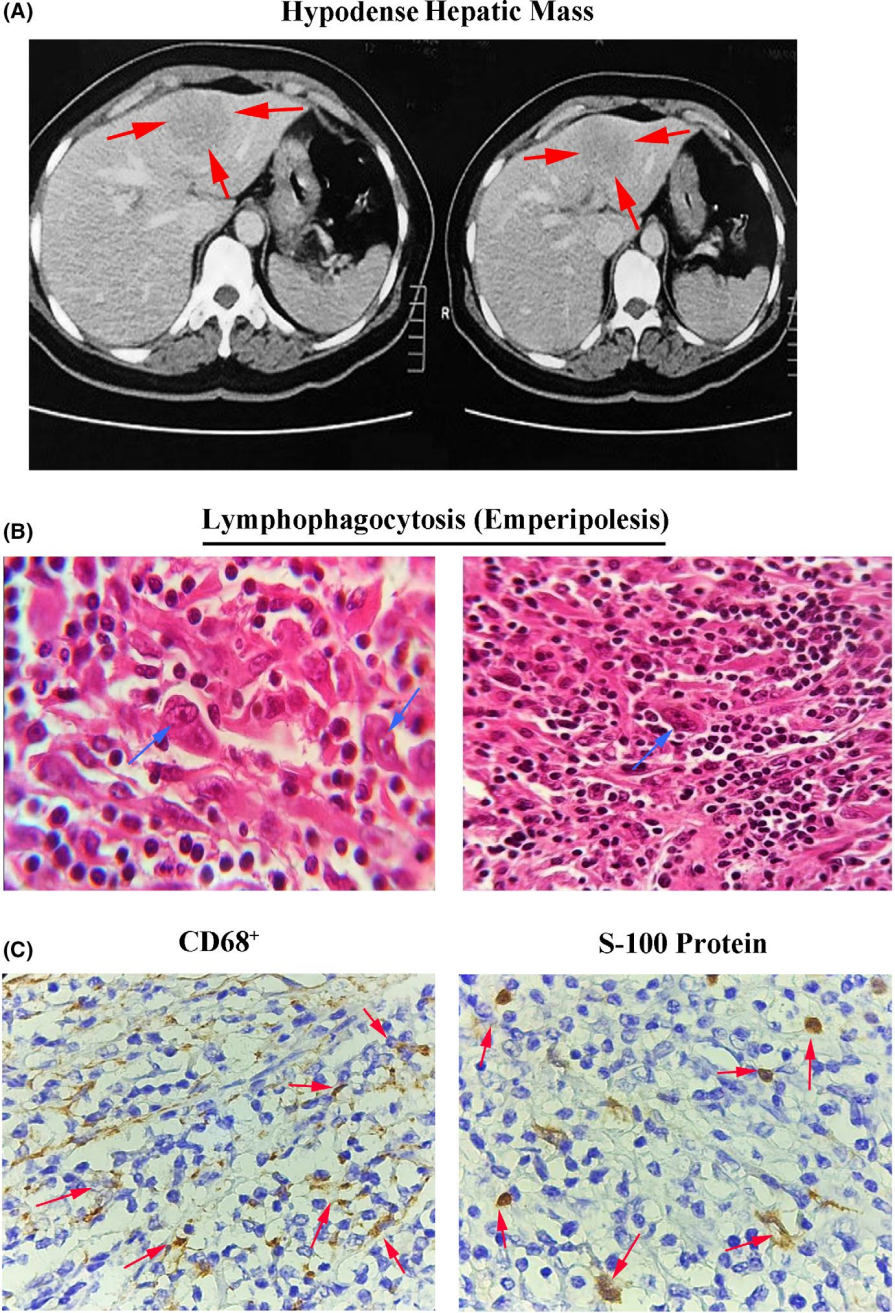
An axial view of non‐enhanced hypodense mass in the left lobe of the liver tissue is represented by computed tomography (CT) scan imaging (A), the representative image of histiocytes with eosinophilic nucleolus stained by hematoxylin and eosin (H & E) method. Some dual or multinucleated occasionally represent a lymphophagocytosis (emperipolesis, left image: x40, right image: x10) (B), histiocytes stained positively for CD68 marker and protein S‐100 with immunohistochemistry (IHC) technique (x40) (C)

A 51‐year‐old woman was admitted to the hospital with chief complaints including chronic abdominal pain, weight loss (5% of body weight), a history of undulant fever, and nocturnal sweating. In physical examination, orthostatic hypotension, conjunctival pallor, and abdominal tenderness of the right upper quadrant were observed. Laboratory tests showed an elevated erythroid sedimentation rate (ESR, 87mm/h) and normocytic normochromic anemia (hemoglobin = 9.3 g/dl). Also, abdominal ultrasound revealed a 37mm×49mm cyst‐like mass in the left lobe of the liver (4A segment) without internal septation. To further ascertain, imaging by computed tomography (CT) scan also showed a hypodense non‐enhancing lesion with a normal margin (4 × 5 cm) in the left lobe of the liver (Figure 1A). We re‐checked the patient's past medical history to find further diagnostic clues for the solitary liver mass. A genetic family history, as one of the crucial risk factors, was also negative for all chronic liver diseases, as well as viral hepatitis infections. In addition, the stigmata of chronic liver disease (eg, cirrhosis) were not identified. Moreover, viral hepatitis panel, alpha‐fetoprotein (AFP), and CA19‐9 were undetectable. Colorectal cancer regular screening tests were appeared to be normal six months before the onset of symptoms.

After that, she was referred to Shahid Ghazi Hospital, Tabriz, for hepatic resection. During postoperative pathological examinations, proliferated histiocytes, eosinophilic nucleolus, and dual or multinucleated monocytes were observed, which exhibited a lymphophagocytosis (emperipolesis) (Figure 1B). It has been also reported that the histiocytes were integrated with a polymorphonuclear leucocytes (polymorphs) infiltration composed of mononuclear and plasma cells. Peripheral blood smear and bone marrow aspiration were normal, while bone marrow biopsy showed lymphoplasmacytosis with negligible megakaryocytosis. For further substantiation, the IHC staining was performed for presumable detection of CD68 marker and S‐100 protein into the histiocytes and CD138, kappa, and lambda markers into the plasma cells. The results showed that the histiocytes were positive for CD68 marker and S‐100 protein (Figure 1C). Differential diagnosis regarding the presence of Hodgkin's lymphoma was also performed by CD15 marker and CD30 marker staining, and subsequently, negative results were achieved. In addition, BRAF^V600E^ mutation was not detected in this report. As a consequence, the hepatic histological paradigm was totally in favor of RDD.

Finally, the patient was transferred to the hematology‐oncology ward to initiate pharmacotherapy with systemic prednisone, 30 mg/day. She was conservatively followed up with consecutive CT imaging revealed no recurrence after 3 months. It has been also established that the patient responded successfully to the corticosteroids therapy. Despite receiving 5 mg/day prednisone, she is consistently followed up once a year.

## 3 DISCUSSION

As sinus histiocytosis with massive lymphadenopathy, RDD is defined as an exceedingly rare non‐Langerhans cell reactive histiocytic disorder preliminarily described in 1969.^8^ The prevalence of RDD is approximately one in 200,000 cases in the United States and commonly present with fever, neutrophilia, increased serum ESR, leukocytosis, lymphopenia, polyclonal hyperglobulinemia, and anemia. However, in most identified cases, there is no apparent or specific symptom.^7^

Nodal RDD, described basically by Destombes and colleagues, is recognized by a sinus expansion of the large histiocytes with “watery‐clear” cytoplasm, as well as a large foamy and prominent nucleus.^4^ Besides, consistent features include the cytomorphology of the large pale histiocytes and their immunophenotype. Emperipolesis, the trafficking of intact leukocytes through the cytoplasm, is another helpful finding but is not required for diagnosis because it can be focal, especially at extranodal sites and may be observed in other histiocytoses such as juvenile xanthogranuloma and malignant histiocytoses. Extranodal lesions are usually associated with more fibrosis, fewer RDD histiocytes, and less emperipolesis. In these cases, immunostaining is required to highlight the residual RDD histiocytes in highly detectable lymphoplasmacytic lymphoma with stromal fibrosis and a variety of xanthogranulomatous responses. The immunophenotype of the large RDD histiocytes is mainly characterized by cytoplasmic and nuclear S‐100 and fascin positivity, as well as CD68^+^, CD163^+^, and CD14^+^ cells. It is worth noting that in contrast to Langerhans cell histiocytosis, these cells are negative for CD1a and CD207 markers.^4^ Although extranodal involvement in RDD is common and may occur in more than 40% of patients without lymphadenopathy occurrence,^6^ intra‐abdominal involvement is uncommon with an incidence of 4%.^9^ Of note, the GI manifestation mostly affects middle‐aged females.^10^ In this line, reported pancreatic or hepatic involvement is extremely rare with more tend to present in younger patients.^11^

In line with our report, pieces of literature also reported RDD occurrence in various organs in a wide range of ages. In 2011, RDD was reported in a 2‐year‐old girl with a progressive CLA associated with persistent fever, which was further confirmed by radiological findings, histopathological, and immunohistochemistry assessments.^12^ Another case report regarding RDD in childhood was also reported by Xu et al.^13^ According to their report, a soft tissue involvement, a benign tumor, was observed in a 17‐month‐old girl following the biopsy of subcutaneous mass without the signs of pain, erythema, and swelling with positive results for S‐100 protein and CD68 marker.^13^ After the surgical intervention and two years following up, no evidence of recurrence or metastasis was reported. As a disseminated form, multiple intracranial involvements were also reported, recently. An adult woman was presented with intractable headaches and a history of RDD who failed to respond to both chemo‐ and radiation therapies.^14^ Following the CT and MRI head scans and pathological confirmation, she underwent the surgical excision. In parallel with this report, in 2020, Rezaei et al. also found an intracranial RDD in an adult man referred to a clinical care center with dizziness, seizure, hemiparesis, and right hemisensory deficit. Specific markers staining revealed that both S‐100 and CD68 were positive, while CD1a marker was negatively reported. Similar to our report, BRAF^V600E^ mutation was not observed.^15^

## 4 CONCLUSION

To our knowledge, this case is the first adult patient that was reported with solitary liver RDD, without lymphadenopathy in which the patient's symptoms alleviated following the corticosteroid therapy. Although a complete remission has not been expected in similar cases, we highly recommend corticosteroid therapy as the first‐line treatment, which can be accompanied by other therapeutic strategies in non‐responsive patients and life‐threatening conditions along with a consecutive imaging and regular follow‐up.

## CONFLICT OF INTEREST

All of the authors report no kind of conflict of interests in this study.

## AUTHOR CONTRIBUTIONS

All of the authors in this study have contributed equally in design, performance, data collection, and writing and review of the manuscript.

## ETHICAL APPROVAL AND CONSENT TO PARTICIPATE

The patient's identity is secret and preserved unknown in the article, and the patient received an oral and written permission form that was approved by the ethics committee of Tabriz University of Medical Sciences and Urmia University of Medical Sciences. The consent was obtained from the study participant prior to study commencement, and the study participant gave consent to publish.

## CONSENT TO PUBLISH

Signed consent for publication has been obtained from the patient and legal guardian.

## Data Availability

Data sharing is not applicable to this article as no datasets were generated or analyzed during the current study.
